# Biophysical, Nutraceutical, and Technofunctional Features of Specialty Cereals: Pigmented Popcorn and Sorghum

**DOI:** 10.3390/foods12122301

**Published:** 2023-06-07

**Authors:** Valery Tixian Robles-Plata, Sergio Serna Saldivar, Juan de Dios Figueroa-Cárdenas, William L. Rooney, Juan Pablo Dávila-Vega, Cristina Chuck-Hernández, Anayansi Escalante-Aburto

**Affiliations:** 1 Escuela de Ingeniera y Ciencias, Tecnologico de Monterrey, Monterrey 64849, Mexico; a01746831@tec.mx; 2 Tecnologico de Monterrey, Centro de Investigación y Desarrollo de Protenas (CIDPRO), Monterrey 64849, Mexico; jpdavila@tec.mx; 3 Centro de Investigación y Estudios Avanzados (CINVESTAV), Unidad Querétaro, Querétaro 76230, Mexico; jfigueroa@cinvestav.mx; 4 Department of Soil and Crop Sciences, Texas A&M University, College Station, TX 77843, USA; wlr@tamu.edu; 5 Tecnologico de Monterrey, The Institute for Obesity Research, Monterrey 64849, Mexico; cristina.chuck@tec.mx

**Keywords:** popcorn, pigmented maize, sorghum, rheological properties, nutraceuticals, cereals

## Abstract

Different pigmented corn and sorghum types were evaluated to characterize their biophysical, nutraceutical, and technofunctional properties for the first time. Commercially pigmented (blue, purple, red, black, and yellow) popcorn (*Zea mays* var. *everta*) and sorghum (*Sorghum bicolor* L.) of yellow and red colors were analyzed. Biophysical and proximal analyses were performed using official methods. The nutraceutical profile included the total phenolic and anthocyanin content. In addition, rheological, structural, and morphological studies were conducted. The results demonstrated significant differences between the popcorn samples and grain types, especially in terms of their biophysical and proximate features. The nutraceutical profile revealed that these specialty grains contained higher concentrations of antioxidant compounds (up to 3-fold when compared with the other grains). The rheological analysis demonstrated that sorghum grains developed higher peak viscosities than popcorn. According to the structural assessments, the type A pattern displayed peaks at the interplanar spaces corresponding to the crystalline and amorphous regions in all the samples. The data obtained in this study provides a base to further investigate the products obtained using these biomaterials.

## 1. Introduction

Producing and consuming nonconventional cereals is considered essential to ensure availability for future inhabitants [1]. Maize (*Zea mays* L.) is one of the most versatile crops worldwide. In recent decades, pigmented varieties from *Cacahuacintle* and popcorn (*Z. mays* var. *everta*) races have been investigated because of their nutritional and antioxidant properties [2,3]. Maize is pigmented mainly due to the presence of a substantial number of secondary metabolites, such as phenolic acids, carotenoids, and anthocyanins, that are chiefly responsible for producing different pigments and can serve as beneficial dietary elements because of their antioxidative properties and potential anti-inflammatory effects. Pigments are primarily concentrated on the thick pericarp or aleurone layers of kernels and corn cobs. Physical qualities, such as grain size, density, hardness, and chemical composition, contribute to the variation in different pigmented corn types; therefore, each variety has a specific observable property [4].

Moreover, after wheat, rice, maize, and barley, sorghum (*Sorghum bicolor* L.) is the most produced cereal worldwide. Because of its outstanding production and tolerance to heat, drought, and pests, this crop is replacing corn in some areas [5]. Sorghum contains high levels of polyphenols, such as phenolic acids, flavonoids, and anthocyanins, that offer various health benefits. In sorghum grains, polyphenols are mainly located in the layers of the pericarp, testa, and aleurone [6]. Moreover, numerous studies have reported that some compounds in sorghum exhibit beneficial effects against the most prevalent human illnesses, such as diabetes, obesity, metabolic disorders, and inflammation [7].

Popcorn and sorghum can be used to obtain second-generation snacks as their grains can be directly expanded by applying heat [8]. The pop-ability of popcorn and other related cereals, such as sorghum, is strongly associated with the physical properties of kernels, especially pericarp thickness and the structure of vitreous endosperm cells [9]. Furthermore, information regarding the biophysical, morphological, and nutraceutical traits of grains is essential for selecting high-yield seeds, which is also associated with economic issues [10]. This is the first study to evaluate the biophysical, nutraceutical, and technofunctional properties of five popcorn and two sorghum varieties. This characterization helps in evaluating future applications to produce functional foods with added value.

## 2. Materials and Methods

### 2.1. Biological Materials

Six commercial types of pigmented popcorn maize (*Z. mays* var. *everta*; blue, purple, red, black, and yellow), purchased from a local market, and two cultivars of yellow and red sorghum (*Sorghum bicolor* L.) were used. The yellow sorghum variety was developed in the experimental fields of Texas A&M and the red sorghum variety was obtained from a local supplier in Queretaro, México. All the samples were purchased in 2022. The grains were cleaned using a 149-µm US sieve No. 100 in order to remove foreign material. The biophysical properties were evaluated using cleaned whole kernels. Grains processed in a grinder with a stainless steel blade (Krups^®^, model F203, Port Orchard, WA, USA) were used for other determinations. Popcorn, sorghum grains, and flours were stored at 5 °C in sealed polyethylene bags until use.

### 2.2. Biophysical Properties

In total, 1000 grains were weighed (g) on an analytical balance (Sartorius, model MCE, Aubagne, France) in triplicate. In addition, the length, width, and thickness (mm) of 30 grains randomly obtained for each type of corn and sorghum were measured using a digital vernier (Leidsany, Britt Technology Inc., Louisville, KY, USA) [11].

### 2.3. Proximal Analysis

The proximal analysis was performed in triplicate according to the following official methods of AOAC. The protein analysis was conducted using the official method 978.02 with a 6.25 conversion factor, the moisture analysis was performed using the gravimetric method 925.10, the ash content was determined using the 923.03 procedure, and the crude fiber content was measured using the 962.09 method [12]. The crude fat content was determined using the AACC method 30–20.01 protocol [13]. The content of the nitrogen-free extract (NFE %) was calculated by difference [14] as follows:

(1) $$NFE\;\left(\%\right)=100-\%Protein-\%Moisture-\%Ash-\%Crude\;fiber-\%Crude\;fat$$

### 2.4. Nutraceutical Properties

#### 2.4.1. Quantification of Total Phenolic Compounds

The Folin–Ciocalteu (FC) test [15] was used to determine the total phenolic content of each extract using methanol as the solvent. The sample extract was obtained by weighing 500 mg of the sample and adding 5 mL of anhydrous methanol (Sigma-Aldrich^®^, 99.8% purity, Burlington, MA, USA). This mixture was vigorously vortexed. Then, the samples were placed in a sonicator (Brandson 5510, Danbury, CT, USA) at 40 kHz for 30 min and centrifuged at 4000 rpm for 30 min. A 100 L aliquot of the sample was placed in a 2 mL microtube. Then, 200 µL of FC reagent at 10% (*v*/*v*) (Phenol Reagent of Folin–Ciocalteu, Sigma-Aldrich^®^, Burlington, MA, USA) was added to the sample and was rigorously vortexed. The mixture was left for 2 min. Then, 800 L of 700 mM Na_2_CO_3_ (Sigma-Aldrich^®^, 99% purity, Burlington, MA, USA) was added to the mixture and incubated for 2 h at room temperature. The total phenolic content was determined at an absorbance of 765 nm using a spectrophotometer (Thermo Scientific, Model Evolution 300, Austin, TX, USA). Gallic acid (Sigma-Aldrich, 98% purity) was used for the calibration curve (0–0.05 mg mL^−1^), and the total phenolic content was calculated and expressed as milligrams of gallic acid equivalents per gram of sample dry weight (mg GAE g^−1^).

#### 2.4.2. Quantification of Total Anthocyanins

Total anthocyanins were examined using a previous method [16] with some modifications. First, 600 mg of the sample was weighed and 4.8 mL of acidified ethanol (AZ^®^, 96%) (ethanol + 1 N HCl, 85:15 *v*/*v*) was added (36.55–38% HCl, J.T. Baker^TM^, Mexico City, Mexico). The mixture was vortexed for 30 min. The tubes were centrifuged at 3000 rpm for 10 min, and the supernatant was obtained. Absorbance was measured at 520 nm using the UV-Vis spectrophotometer (Thermo Scientific, Model Evolution 300, Fitchburg, WI, USA). Cyanidin-3-glucoside (kuromanin chloride analytical standard, ≥98% purity, Merk^®^, Darmstadt, Germany) was used as a standard pigment. A series of standard solutions of cyanidin-3-glucoside was prepared at 0–0.02 mmol (0–27 μg/mL). Data were expressed as milligrams of cyanidin-3-glycoside equivalents per kilogram of dry weight (mg C3G kg^−1^).

### 2.5. Rheological Assessment

Rheological analyses were performed using 4 g of each sample. The sample was conditioned to a moisture content of 14% and weighed in an aluminum can. Then, 24 mL of distilled water was added. The dispersed solution was stirred with a plastic paddle, and a heating cycle was started at a controlled rate (50° C–90 °C–50 °C). The maximum viscosity, final viscosity, retention force, breakdown, and setback in cP were analyzed, and the rheological behavior was analyzed using a Rapid Visco Analyser (model RVA-3D, Newport Scientific, Warriewood, Australia) [17].

### 2.6. Structural Properties (X-ray Diffraction)

The milled grain samples were adjusted to a moisture content of 7% according to Escalante-Aburto et al.’s procedure [17]. A structural property analysis was conducted using an X-ray diffractometer (DMAX-2100, Rigaku, Tokyo, Japan), which was operated under the following conditions: 30 kV and 16 mA with CuKa radiation of λ = 1.5405. The flour samples were scanned from 5° to 50° on the 2θ scale. The Bragg equation was used to calculate the interplanar spacing (*d*) of the peaks.

### 2.7. Morphological Assessments

Grain morphology was examined using a scanning electron microscope (Phillips model XL30) at an accelerating 20 kV (50 mA) voltage. The grains were segmented longitudinally using a razor blade and were fixed over an aluminum base. Images were captured at 500×, 1500×, and 5000× magnification from the hard, intermediate, and soft endosperm and the protein bodies within the matrix.

### 2.8. Design of Experiments and Statistical Analysis

A completely randomized design of experiments was used. All the determinations were performed in triplicates, and the means and standard deviations (SDs) were reported. A one-factor ANOVA and a mean comparison analysis (Tukey test) were performed with 95% confidence. Both analyses considered *p* < 0.05 to be of statistical significance. Minitab^®^ LLC (State College, PA, USA), statistical analysis software version 21.4.0.0 was used.

## 3. Results and Discussion

### 3.1. Biophysical Properties

Table 1 and Table 2 present the biophysical parameters of the popcorn and sorghum cultivars. As expected, significant differences were observed between the popcorn and sorghum cultivars because of their botanical and agronomic origins.

Among the popcorn varieties, the blue grains presented with the highest 1000-grain weight value. All the other corn samples exhibited significantly lower values, but the black grains had the lowest values. Both sorghum samples exhibited significantly lower 1000-grain weight values than the popcorn samples. Yellow and red sorghum had the same 1000-grain weight, and no significant differences were noted (Table 2).

The values for the geometrical measurements were similar among the popcorn samples. Nevertheless, significant differences were observed in length, where blue, purple, and yellow popcorn exhibited the highest values of length. The yellow popcorn exhibited the highest width. The blue and purple samples presented the highest values of thickness. Tian et al. [18] evaluated three popcorn varieties of yellow commercial grains and found that the size of the kernel was highly associated with their densities and expansion volume.

No statistical differences were observed in the geometrical features of the sorghum varieties. However, the physical features of the grains also account for their engineering properties, and the information they provide is essential for designing specialized equipment and for determining their behavior during handling and processing. Surpam et al. [19] evaluated the engineering properties of sorghum (Rabi Jowar) and reported average values of 4.3, 4.2, and 2.6 mm for length, width, and thickness, respectively, which are similar to the values reported for our samples.

### 3.2. Proximal Analysis

Table 3 and Table 4 present the results of the proximal assessments of popcorn and sorghum cultivars. The moisture content of black popcorn was the lowest compared with those of other popcorn grains and was similar to those of sorghum grains. No significant differences in moisture content were observed between the samples. All the grain samples were stored at adequate relative moisture conditions in order to avoid microbial spoilage [20]. The average protein content of the popcorn samples was 11.90%, and no significant differences were reported in protein content. These protein content values are similar to those reported by Farahnaky et al. [21] for two popcorn genotypes (hybrid KSC 600PC and another from a local market). They reported protein contents of 12.4% and 11.4%, respectively. Yellow sorghum grains exhibited protein content levels similar to those of the popcorn varieties; red sorghum exhibited the lowest protein content (almost 45% less protein). In fact, the protein content of both sorghum types was significantly lower than that of four African samples (white, yellow pale, yellow, and red), accounting for an average protein content of 22.53% in dry weight [22].

Nevertheless, the moisture content of the sorghum samples used in this study was significantly higher than that of an Indian variety (9.25%) [19]. The lipid content ranged from 2.96% to 4.11% for the popcorn samples and 2.57% to 3.34% for the sorghum grains. The fat content of our samples was significantly higher than those reported by Palavecino et al. [23] in commercial samples from Central Argentina. They reported fat concentrations of up to 5.73% in hybrid sorghum cultivated in Argentina (Pioneer-80T25).

No significant differences in ash content were observed among the grain types and cultivars. The blue, purple, and black popcorn exhibited the highest crude fiber content, but the yellow sorghum variety significantly differed from the red variety in crude fiber content. The sorghum varieties presented the highest NFE values (71.16%) compared with all the popcorn samples, which had NFE values ranging from 64.68% to 68.02%.

The crude fat and ash contents of the sorghum grains were in the same magnitude as those of a white sorghum *Paloma* variety reported by Hernández-Becerra et al. [24]. They reported crude fat and ash contents of 2.7% and 1.3%, respectively. According to Khalid et al. [7], the total carbohydrate content of sorghum is between 57% and 80.6%, and this result is similar to our findings.

### 3.3. Nutraceutical Characteristics

**Figure 1:**
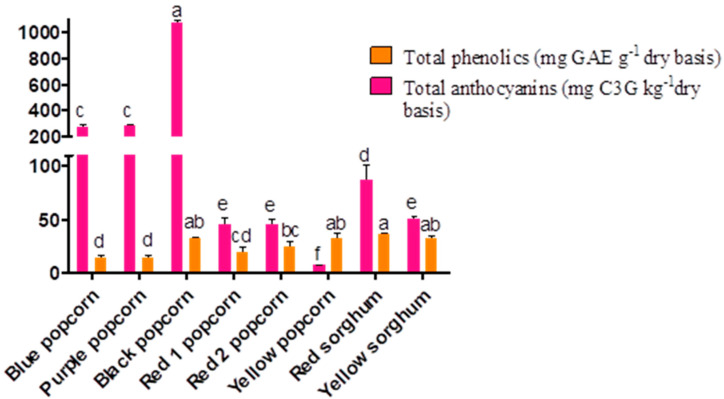
Functional properties of popcorn and sorghum grains: phenolic and anthocyanin content. Different letters represent statistical differences at *p* < 0.05. The bars indicate the SD.

The nutraceutical properties evaluated in the specialty grains are shown in Figure 1.

The anthocyanin content of all the popcorn samples ranged from 6.9 to 1073.5 mg C3G kg^−1^, and black popcorn presented the highest concentration of this antioxidant molecule. The average anthocyanin content of popcorn and sorghum was 287.41 and 69.03 mg C3G kg^−1^, respectively. Among all the samples, red sorghum had the highest anthocyanin content. However, this value was lower than that reported by Xu et al. [25] for red pericarp sorghums (2660–8930 mg C3G kg^−1^). On the other hand, the highest total phenolic content was observed in the black and yellow popcorn samples. The total phenolic contents of the blue, purple, and red 1 popcorn samples were lower. The total phenolic contents varied from 32.34 to 13.80 mg GAE g^−1^ for the popcorn samples. Our popcorn samples exhibited a higher total phenolic content than Iranian popcorn (*Z. mays* var. *everta*) grains (0.13 mg GAE g^−1^) [26]. These assessments in raw popcorn samples are crucial because some studies have suggested that the total phenolic content and antioxidant capacity (evaluated by FRAP) of pigmented raw and popped kernels do not differ significantly [27].

Both sorghum varieties exhibited the same total phenolic content; the average total phenolic content was 34.27 mg GAE g^−1^. However, the red sorghum grains exhibited a higher concentration of total phenolics than the yellow sorghum grains. The total phenolic content of our sorghum samples was higher than those reported by Bhukya et al. [28] in 60-grain sorghum genotypes, including white, red, and brown pericarps. They reported a total phenolic content of 0.05–4.23 GAE g^−1^. The total phenolic content in our samples was up to 3-fold higher than that reported in six sorghum varieties of brown, red, and white pericarps (11.50–0.24 mg GAE g^−1^) [29]. Similarly, the total polyphenol content of red sorghum purchased from Maroua, Cameroon (82.2 mg GAE g^−1^) [22] was higher than that of our study samples. In addition to the agronomical, harvesting, and storage conditions of the samples, extraction procedures (refluxing, maceration, milling, and solvent types) commonly influence the total phenolic and anthocyanin content in these grains. Regarding the milling factor, Rumler et al. [30] evaluated dried and dehusked red sorghum (variety *Armorik*, obtained in Austria) and reported total phenolic contents of 152.2, 237.2, and 155.5 mg FAE 100 g^−1^ in whole sorghum flours obtained using a dry-flake squeezer, a pilot-scale stone mill, and an industry-scale roller mill, respectively. The particle size and milling process remarkably affects the extraction rate of these compounds. Furthermore, during extraction, some pericarp remains intact in the flour. Thus, additional studies must be conducted to avoid underestimation when quantifying these compounds.

### 3.4. Rheological Behavior

**Figure 2:**
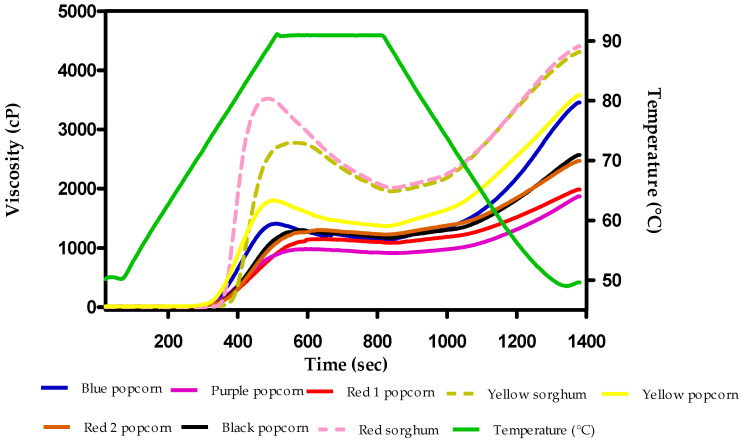
Rheological behavior of the evaluated popcorn and sorghum grains.

Figure 2 presents the rheological behavior of raw grains from the popcorn and sorghum varieties. The sorghums presented higher viscosity than all the popcorn samples. The yellow sample exhibited the highest peak viscosity among all the popcorn samples. Both sorghum varieties exhibited higher values than the popcorn lines for all the parameters evaluated in the viscoamylographic analysis, except for the breakdown parameter (Table 3).

Table 5 and Table 6 list the specific parameters of rheological behavior (RVA profile). The sorghum lines exhibited higher peak viscosities than the popcorn varieties, which was expected because, at this stage of the RVA analysis, the hydration of starch molecules occurred. According to the proximal analysis results, the sorghum grains contain more starch than the popcorn samples (Table 6). The peak viscosity values increased due to increased interactions between water molecules and starch granules during gelatinization. In this context, the peak viscosities of both sorghum varieties differed from those reported by Sang et al. [31]. They reported peak viscosities of 2750, 1750, and 1250 cP for evaluated waxy, heterowaxy, and normal sorghum hybrids, respectively. This indicated that the varieties used in the present study had some characteristics of waxy endosperm; hence, they contained less amylose content. Consequently, the breakdown values of the sorghum samples were considerably higher than those of all the popcorn samples. Breakdown is related to the differences in the type of starch and its structure, such as granule rigidity and crystallinity, as well as the amylose and lipid content [32]. The lower breakdown values of popcorn samples could also be attributed to increased interactions between starch and protein. These interactions make the starch granules rigid and reduce the susceptibility of grains to breakage during heating and shearing cycles [33]. Consequently, the breakdown values of the popcorn samples used in the present study differed from those of the yellow popcorn samples analyzed by Paraginski et al. [33]. They reported breakdown values between 421.5 and 91 cP.

Melting of the crystalline regions increases the movement of water molecules inside the starch granules. This represents holding strength. Holding strength is the lowest viscosity value measured at the end of the heating cycle (holding stage) [32]. The blue and yellow popcorn grains exhibited the highest breakdown values. These popcorn grains likely have a larger proportion of vitreous endosperm cells, which increases the breakdown values (184 and 431 cP, respectively). This can be attributed to the compactness of the endosperm, which prevents all the water molecules from gelatinizing all the starch granules. The sorghum samples exhibited significantly higher holding strength values. The holding strength values of our samples were lower than those of 13-grain sorghum varieties cultivated in New South Wales (mean: 2969 cP). Nevertheless, some varieties exhibited similar holding strength results, which could be related to chemical interactions between kafirin and phenolic contents with starch. These interactions require further investigation in future studies [34].

Concerning the setback (SB) region, the blue and black popcorn grains presented significantly higher values. This parameter represents the increase in viscosity when the cooling stage ends and starch retrogradation occurs. The leached amylose and amylopectin chains form a new crystalline structure through realignment [32]. Thus, these popcorn grains could have more intergranular interactions in the suspension, such as entanglement between the surface particles of the adjacent starch granules, thereby increasing the viscosity values [35]. The sorghum grains presented lower SB values than those reported by Truong et al. [34]. They also found a significant relationship between SB and the ferulic acid content in sorghum kernels. The final viscosities in the red and yellow sorghum samples were not of the same magnitude as those reported in 20 high-yield sorghum hybrids harvested in South America. These sorghum hybrids had white, red, and brown pericarps, and their final viscosities ranged between 3030 and 4402 mPa s [23].

### 3.5. Structural Properties (X-ray Diffraction)

**Figure 3:**
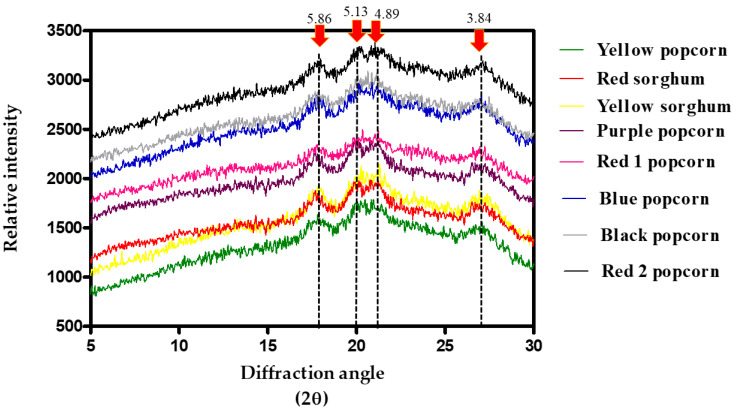
X-ray diffraction analysis of the evaluated nonconventional grains of sorghum and corn.

Figure 3 depicts the diffractograms obtained from the specialty cereals of popcorn and sorghum. The typical structure of X-ray diffraction patterns for the popcorn lines and sorghum varieties was type A, which corresponds to native starches found in cereals [36].

Four peaks corresponding to the crystalline structure of native starch granules were detected [36]. Strong reflections can be observed at 17.5° and 27.5° (2θ), corresponding to the interplanar spaces (d) of 5.86 and 3.84, respectively. Furthermore, a doublet at 20.5° and 21° (2θ) was detected at the interplanar spaces of 5.13 and 4.89 (d), which agrees with the structures previously reported for the whole grain flours of corn and sorghum [37,38]. No differences were observed in the X-ray diffraction patterns of the popcorn and sorghum lines. Nevertheless, yellow sorghum exhibited more defined peaks than red sorghum. The findings of the popcorn patterns were the same as those reported by Trovo et al. [39] in starch extracted from creole popcorn grains, with peaks observed at 15°,18°, 19°, and 23° at 2θ.

### 3.6. Morphological Assessments

**Figure 4:**
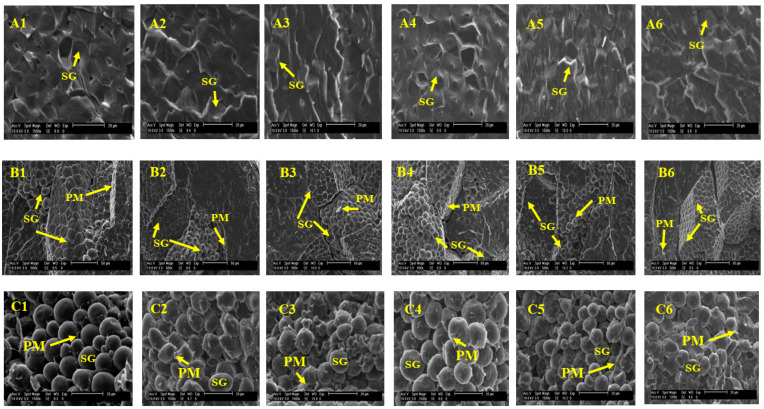
Micrographs of the hard (**A**) at 1500×, intermediate (**B**) at 500×, and soft endosperm (**C**) at 1500× of popcorn samples corresponding to (1) yellow popcorn, (2) purple popcorn, (3) red 1 popcorn, (4) blue popcorn, (5) black popcorn, and (6) red 2 popcorn. Abbreviations: SG, starch granules; PM, protein matrix.

**Figure 5:**
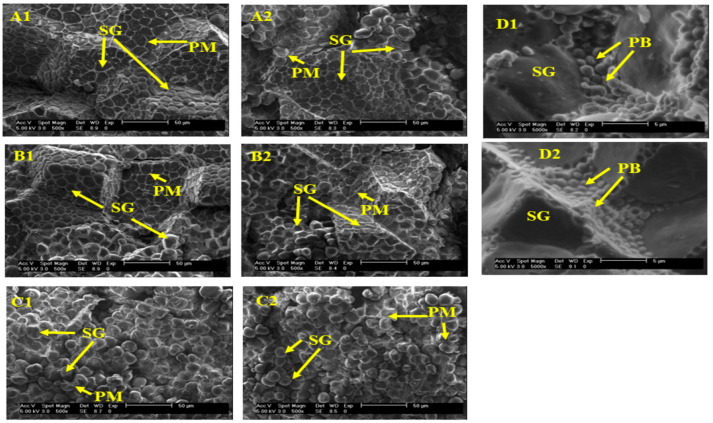
Micrographs of the hard endosperm (**A**) at 500×, intermediate endosperm (**B**) at 500x, soft endosperm (**C**) at 5000×, and (**D**) protein bodies at 5000× of sorghum samples corresponding to (1) red and (2) yellow sorghum. Abbreviations: SG, starch granule; PM, protein matrix; PB, protein bodies.

Figure 4 and Figure 5 present the micrographs of the popcorn and sorghum samples, respectively, exhibiting their soft, hard, and intermediate endosperms. Figure 4(A1–A6) presents the polyhedral structure with angular shapes of the starch granules found in the hard endosperm of popcorn grains. The size of the starch granules in these grains was approximately 10 µm. The hard and compact structures in our samples are similar to those reported by Singh et al. [35], who isolated starch granules from popcorn samples of different grain sizes. The protein matrix (PM) covered the starch granules (Figure 4(B1–B6)) in the intermediate endosperm. Surrounding the oval starch granules, the protein matrix formed the soft endosperm structures (Figure 4(C1–C6)). In the soft endosperm, the size of the starch granules was approximately 10–15 µm and was similar in all the popcorn samples. The starch structure was intact because the images taken were of half-cut grains and not flour.

According to Ziegler et al. [40], popcorn grains contain 27% amylose and 73% amylopectin, and the form of the granules is spherical and polyhedral, which agrees with the morphological structures of our samples. In the soft endosperm (Figure 4(C1–C6)), some voids were observed among the granules, which are necessary for the arrangement of this grain proportion. This could be related to the expansion volume because samples of progenies with high expansion volumes had a higher proportion of compact endosperm and few voids interspersed among the granules [41]. This should be discussed as a quality parameter for popcorn in further studies.

Although no significant differences were observed in the protein content of popcorn grains, sample red 1 (Figure 4(C3)) exhibited a denser PM covering the soft endosperm. In general, the micrographic images of popcorn kernels agree with the maize type and findings reported by Cui et al. [42]. Furthermore, electron microscopy images of opaque and translucent endosperm in a popcorn hybrid from Iran and American popcorn agree with those of our samples. They demonstrate the presence of polygonal (pentagonal and hexagonal) starch granules in the translucent endosperm (hard), with the starch granules measuring 15 m in size. These structures are closely packed with no spaces among them [21], as observed in Figure 4(B1–B4). During heating, the vitreous (translucent) proportion exhibits the melting of the crystalline regions and gelatinization because water vapor is forced into the starch granules, causing further expansion [41].

Figure 5(A1,B1,C1) presents the hard, intermediate, and soft endosperm of the red sorghum grains, respectively. The compact structure of the starch granules was observed in the hard proportion (A1) with the PM cover, even when the protein content of this type of grain was the lowest (7.93%). The size of the starch granules was ~10–25 µm, and the granules had a polyhedral shape and defined limits, contrary to the structures found in the popcorn lines. Figure 5(D1) depicts the presence of multiple indentations on the grain surface caused by protein bodies and concurs with the morphological descriptions of the vitreous endosperm of sorghum lines reported by Bean et al. [43].

A study conducted on sorghum grains and changes in their morphology reported similar findings about the morphological properties of these grains using the same methodology. The shape and size of the starch were similar (i.e., polyhedral and spherical) in the hard and soft endosperm sections [24]. Evaluating the endosperm type and proportion in grains used for expanded products is important because the vitreous endosperm is the structure that primarily contributes to kernel expansion during popping [41].

## 4. Conclusions

This is the first study analyzing the properties of pigmented specialty popcorn and sorghum grains. According to their agronomical origin, popcorn and sorghum varieties exhibited different biophysical properties. These experiments were performed using commercial batches of specialty grains, and the results may vary when new batches are analyzed. This is expected because these biomaterials might vary during each harvesting season. Nevertheless, the first characterization of new bioproducts is always necessary for understanding more complex behaviors when the biomaterials are processed into final products. The physicochemical traits of both the grain types and varieties were similar to those given in the literature. Nevertheless, in terms of the nutraceutical features, the sorghum samples exhibited higher total phenolic content than the other varieties. Moreover, the total anthocyanin content of the red, purple, blue, and black popcorn was extremely high compared with that of the commercial grain, which was expected because of their nature. The same trend was observed in the red sorghum variety. The rheological, structural, and morphological assessments revealed consistency with the characteristics reported in other studies; however, the content of nutraceutical compounds (phenolics and anthocyanins) can influence these properties. In general, these grains have the potential to be used as functional ingredients and could be added in the cereal industry to produce low-caloric or low-glycemic foods. Further studies are required to investigate this. Full characterization of these specialty grains is crucial because these characteristics influence the functional and technological properties of their final products and derivatives.

## Figures and Tables

**Table 1:** Biophysical properties of different popcorn grains.

Sample	1000 Grains Weight (g)	Length (mm)	Width (mm)	Thickness (mm)
Blue	163.757 ±1.43 ^a^	8.66 ± 0.41 ^ab^	5.46 ± 0.36 ^b^	4.34 ± 0.46 ^a^
Purple	136.16 ± 2.32 ^c^	8.79 ± 0.61 ^a^	5.41 ± 0.57 ^b^	4.21 ± 0.43 ^ab^
Black	95.77 ± 1.82 ^e^	8.07 ± 0.61 ^c^	4.86 ± 0.42 ^d^	3.96 ± 0.53 ^bc^
Red 1	124.33± 3.14 ^d^	8.40 ± 0.58 ^bc^	5.20 ± 0.44 ^bc^	3.65 ± 0.36 ^c^
Red 2	123.84 ± 0.71 ^d^	8.25 ± 0.40 ^c^	5.00 ± 0.41 ^cd^	3.67 ± 0.37 ^c^
Yellow	158.16 ± 1.06 ^b^	8.71 ± 0.49 ^ab^	5.89 ± 0.49 ^a^	3.95 ± 0.44 ^bc^

Average values ± SD. Different letters in the same column are significantly different at *p* < 0.05.

**Table 2:** Biophysical properties of the sorghum grain types.

Sample	1000 Grains Weight (g)	Length (mm)	Width (mm)	Thickness (mm)
Red	25.18 ± 0.31 ^a^	4.12 ± 0.37 ^a^	3.53 ± 0.22 ^a^	2.55 ± 0.21 ^a^
Yellow	25.52 ± 0.21 ^a^	4.11 ± 0.22 ^a^	3.40 ± 0.24 ^b^	2.53 ± 0.21 ^a^

Average values ± SD. Different letters in the same column are significantly different at *p* < 0.05.

**Table 3:** Physicochemical properties of different pigmented popcorn grains.

Sample	Moisture	Protein	Crude fat	Ash	Crude Fiber	NFE
% (Dry Basis)						
Blue	12.69 ± 0.30 ^abc^	11.08 ± 0.28 ^a^	3.00 ± 0.59 ^bc^	1.60 ± 0.26 ^a^	4.51 ± 0.21 ^ab^	67.08 ± 0.79 ^bc^
Purple	13.06 ± 0.12 ^ab^	11.25 ± 0.15 ^a^	3.79 ± 0.12 ^ab^	1.46 ± 0.23 ^a^	3.98 ± 0.55 ^abc^	66.44 ± 0.15 ^bc^
Black	11.67 ± 0.21 ^cd^	11.91 ± 0.34 ^a^	2.96 ± 0.65 ^bc^	1.53 ± 0.23 ^a^	3.88 ± 0.24 ^abc^	68.02 ± 1.17 ^b^
Red 1	13.76 ± 0.31 ^a^	13.12 ± 0.33 ^a^	3.79 ± 0.13 ^ab^	1.83 ± 0.25 ^a^	2.16 ± 0.11 ^e^	65.29 ± 0.71 ^bc^
Red 2	13.16 ± 0.92 ^ab^	12.94 ± 0.20 ^a^	4.11 ± 0.21 ^a^	1.66 ± 0.30 ^a^	3.48 ± 0.35 ^cd^	64.68 ± 1.23 ^c^
Yellow	12.55 ± 0.01 ^bc^	11.15 ± 2.24 ^a^	3.87 ± 0.48 ^ab^	1.73 ± 0.05 ^a^	3.59 ± 0.23 ^bc^	67.08 ± 1.83 ^bc^

Average values ± SD. Different letters in the same column are statistically different at *p* < 0.05. Abbreviation: NFE, nitrogen-free extract.

**Table 4:** Physicochemical properties of the two types of sorghum grains.

Sample	Moisture	Protein	Crude Fat	Ash	Crude Fiber	NFE
% (Dry Basis)						
Red	11.11 ± 0.38 ^a^	7.93 ± 0.24 ^b^	2.57 ± 0.11 ^b^	1.53 ± 0.05 ^a^	2.61 ± 0.48 ^b^	74.21 ± 0.85 ^a^
Yellow	11.22 ±0.03 ^a^	10.95 ± 0.11 ^a^	3.34 ± 0.22 ^a^	1.53 ± 0.05 ^a^	4.80 ± 0.22 ^a^	68.12 ± 0.19 ^b^

Average values ± SD. Different letters in the same column are statistically different at *p* < 0.05. Abbreviation: NFE, nitrogen-free extract.

**Table 5:** Rheological parameters (RVA analysis) of different popcorn grains.

Sample	Peak Viscosity	Breakdown	Holding Strength	Setback Region	Total Setback	Final Viscosity
			cP			
Blue	1445 ± 7 ^b^	184 ± 6 ^b^	1261 ± 13 ^b^	2929 ± 87 ^a^	3113 ± 93 ^a^	4374 ± 82 ^a^
Purple	965 ± 12 ^f^	55 ± 12 ^c^	909 ± 6 ^e^	969 ± 15 ^e^	1024 ± 9 ^e^	1933 ± 4 ^d^
Black	1261 ± 10 ^d^	58 ± 8 ^c^	1203 ± 3 ^c^	2287 ± 96 ^b^	2345 ± 91 ^b^	3548 ± 88 ^b^
Red 1	1135 ± 8 ^e^	47 ± 8 ^c^	1088 ± 1 ^d^	867 ± 16 ^e^	914 ± 92 ^e^	2002 ± 9 ^d^
Red 2	1304 ± 1 ^c^	68 ± 7 ^c^	1236 ± 6 ^b^	1551 ± 15 ^d^	1618 ± 14 ^d^	2855 ± 14 ^c^
Yellow	1834 ± 12 ^a^	431 ± 31 ^a^	1404 ± 23 ^a^	1738 ± 54 ^c^	2168 ± 22 ^c^	3572 ± 45 ^b^

Average values ± SD. Different letters in the same column are statistically different at *p* < 0.05.

**Table 6:** Rheological parameters (RVA analysis) of two types of sorghum grains.

Sample	Peak Viscosity	Breakdown	Holding Strength	Setback Region	Total Setback	Final Viscosity
			cP			
Red sorghum	3564 ± 12 ^a^	1502 ± 3 ^a^	2061 ± 12 ^a^	833 ± 25 ^b^	2335 ± 23 ^a^	4397 ± 30 ^a^
Yellow sorghum	2750 ± 9 ^b^	778 ± 11 ^b^	1972 ± 4 ^b^	1551 ± 6 ^a^	2329 ± 50 ^a^	4301 ± 4 ^b^

Average values ± SD. Different letters in the same column are statistically different at *p* < 0.05.

## Data Availability

The data used to support the findings of this study can be made available by the corresponding author upon request.
